# Doxycycline plus streptomycin versus ciprofloxacin plus rifampicin in spinal brucellosis [ISRCTN31053647]

**DOI:** 10.1186/1471-2334-6-72

**Published:** 2006-04-11

**Authors:** Emine Alp, Rahmi Kemal Koc, Ahmet Candan Durak, Orhan Yildiz, Bilgehan Aygen, Bulent Sumerkan, Mehmet Doganay

**Affiliations:** 1 Department of Infectious Diseases and Clinical Microbiology, Faculty of Medicine, Erciyes University, Kayseri, Turkey; 2 Department of Neurosurgery, Faculty of Medicine, Erciyes University, Kayseri, Turkey; 3 Department of Radiology, Faculty of Medicine, Erciyes University, Kayseri, Turkey; 4 Department of Microbiology, Faculty of Medicine, Erciyes University, Kayseri, Turkey

## Abstract

**Background:**

The optimal treatment regimen and duration of the therapy is still controversial in spinal brucellosis. The aim of this study is to compare the efficacy, adverse drug reactions, complications and cost of ciprofloxacin plus rifampicin versus doxycycline plus streptomycin in the treatment of spinal brucellosis.

**Methods:**

The patients diagnosed as spinal brucellosis between January 2002 to December 2004 were enrolled into the study. Patients were enrolled into the two antimicrobial therapy groups (doxycycline plus streptomycin vs. ciprofloxacin plus rifampicin) consecutively. For the cost analysis of the two regimens, only the cost of antibiotic therapy was analysed for each patient.

**Results:**

During the study period, 31 patients with spinal brucellosis were enrolled into the two antimicrobial therapy groups. Fifteen patients were included in doxycycline plus streptomycin group and 16 patients were included in ciprofloxacin plus rifampicin group. Forty-two levels of spinal column were involved in 31 patients. The most common affected site was lumbar spine (n = 32, 76%) and involvement level was not different in two groups. Despite the disadvantages (older age, more prevalent operation and abscess formation before the therapy) of the patients in the ciprofloxacin plus rifampicin group, the duration of the therapy (median 12 weeks in both groups) and clinical response were not different from the doxycycline plus streptomycin. The cost of ciprofloxacin plus rifampicin therapy was 1.2 fold higher than the cost of doxycycline plus streptomycin therapy.

**Conclusion:**

Classical regimen (doxycycline plus streptomycin), with the appropriate duration (at least 12 weeks), is still the first line antibiotics and alternative therapies should be considered when adverse drug reactions were observed.

## Background

Human brucellosis is a worldwide zoonosis and affects over 500 000 people yearly in non-industrialized countries of the world. It is still public health problem in most countries of Mediterranean, including Turkey, Balkan, the Middle East and Central and South America [1].

Brucellosis is a systemic disease and may involve any organ system. However, osteoarticular involvement is the most common complication of brucellosis and the reported incidence is 10–85% in most series [2-7]. Spinal column is one of the most frequently affected site in osteoarticular brucellosis. However its incidence is highly variable (2–54%) in different studies, depending on the study population, the species of *Brucella *involved and the difficulty of diagnosis [3-8]. On the other hand, the optimal treatment regimen and duration of the therapy is still controversial in osteoarticular involvement including spinal brucellosis. The combination of doxycycline with streptomycin is accepted to be the most effective regimen in spinal brucellosis [1]. However, therapeutic failure and relapse are still common. The risk of adverse drug reactions is increased due to the prolonged use of these antibiotics and limited the therapy [4,9,10]. Other treatment option, the combination of rifampicin and doxycycline, are associated with worst outcomes [11,12]. In recent years, fluoroquinolones has been shown to be effective both in vitro and in vivo in the treatment of brucellosis [13-19]. Furthermore, due to ability of fluoroquinolones to penetrate and achieve increased concentration in the tissues and their proven efficacy in bone and soft tissue infections, the outcome may be better in spinal brucellosis. The regimens including fluoroquinolones may be an alternative therapy to doxycycline and streptomycin.

The aim of this study is to compare the efficacy, adverse drug reactions, complications and cost of ciprofloxacin (C) plus rifampicin (R) versus doxycycline (D) plus streptomycin (S) in the treatment of spinal brucellosis.

## Methods

This study was planned as an open, controlled and non-randomized. The Ethical Committee of Erciyes University approved the study protocol used in this study. The patients also accepted to take place in this study. The patients diagnosed as spinal brucellosis between January 2002 to December 2004 were enrolled into two study groups consecutively. Age less than 16 years, pregnancy, neurobrucellosis, previous history of brucellosis and antimicrobial therapy and discontinuation of the therapy for any reason (allergy to any of the drugs, death, adverse reactions) were exclusion criteria from the study.

Patients were enrolled into the two antimicrobial therapy groups (DS vs. CR). Patients in DS group received doxcycline, 100 mg twice a day orally for at least 12 weeks, plus streptomycin, 1 g per day intramuscularly for 21 days (1 g every other day for patients more than 60 years of age for 21 days). Patients in CR group received ciprofloxacin, 500 mg twice a day orally, plus rifampicin, 600 mg per day in a single morning dose for at least 12 weeks. The antibiotic therapy was prolonged according to clinical improvement and the resolution of magnetic resonance imaging (MRI) findings. The antibiotic therapy was changed if the adverse effects of the drugs or therapeutic failure were observed. If patient's complaints and findings were partially improved and there was not an increase in MRI findings at the end of 12 weeks, the therapy was prolonged until resolving MRI findings. At least two weeks bed rest was suggested to all patients and lumbosacral orthosis was suggested to patients with pain due to instability. Furthermore, the patients were followed up until 12 month after cessation of the therapy for the evaluation of sequelae and relapse. For the cost analysis of the two regimens, only the cost of antibiotic therapy was analysed for each patient.

### Clinical and laboratory evaluation

The patients were evaluated initially, at the end of the first and second weeks, and monthly for six months and one year after the therapy by two of the authors (EA and RKK). Patients were examined for the therapeutic efficacy and signs of drug toxicity by clinical data and laboratory studies; haemogram, differential leucocyte counts, erythrocyte sedimentation rate (ESR), glucose, blood urine nitrogen (BUN), creatinine, alanine aminotransferase, aspartate aminotransferase, alkaline phophatase, bilirubin, albumin, total protein, electrolyte levels, C-reactive protein (CRP), standard tube agglutination test for *Brucella *and blood culture. At least two blood samples for culture were taken initially.

### Radiographic studies

**Figure 1:**
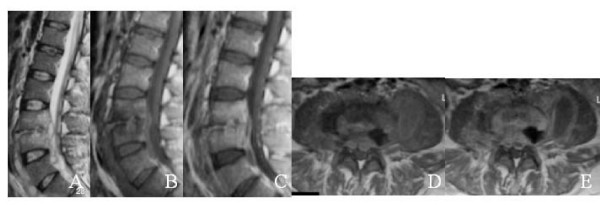
A) T2-weighted image shows increased signal intensity intervertebral disc space with epidural and prevertebral abscess. B) T1-weighted image shows irregularity and destruction of vertebral end plates with iso-to hyperintence signal intensity. C) T1-weighted image after contrast injection shows increased signal intensity intervertebral disc space and the inferior and superior end plates of vertebral bodies. D) T1-weighted axial image shows epidural and paravertebral abscess with iso-to hyperintence signal intensity. E) T1-weighted axial image after contrast injection shows increased signal intensity epidural and paravertebral abscess

**Figure 2:**
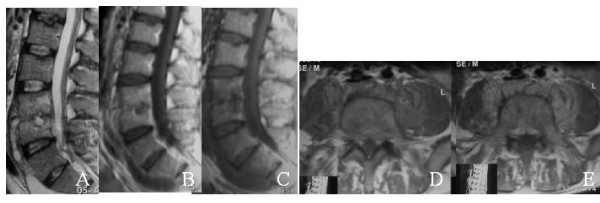
A) T2-weighted image shows increased signal intensity involving subchondral part of L4 spine adjacent to L3-4 disk and decreased signal intensity intervertebral disc space. B) T1-weighted image shows iso-to hypointence signal intensity. C) T1-weighted image after contrast injection shows decreased signal intensity intervertebral disc space. D) T1-weighted axial image shows paravertebral irregulary mass with iso-to hyperintence signal intensity. E) T1-weighted axial image after contrast injection shows increased signal intensity paravertebral mass.

All patients underwent MRI at the beginning of the treatment and end of the treatment. MRI findings for brucellosis were decreased signal intensity in the vertebral bodies on T1-weighted images, increased signal in the vertebral bodies on T2-weighted images, increase in signal in discs on T2 weighted images, loss of end-plate definition on T1 weighted images and contrast enhancement in the discs on T1-weighted images with godolinuium (figure 1) [20]. Improvement was defined as lack of contrast enhancement in the disc space (figure 2).

### Microbiological studies

For agglutination tests, antigen of *B. abortus *S99 obtained from Pendik Animal Diseases Research Institute (Istanbul, Turkey) was used. A positive Wright test was taken as a titer of 1/160 or greater. Blood culture specimens were cultured by BACTEC 9240 system (Becton-Dickinson, Mayland USA). Samples of blood were inoculated into BACTEC system for 14 days. The isolates of Gram-negative cocco-bacilli were identified by conventional biochemical tests (e.g. motility, oxidase, catalase, glucose fermentation, production of H_2_S and urease) and also by an agglutination test using antiserum obtained from patients showing high titer of agglutination. Further identification of *Brucella *strains was not performed.

### Definitions

Brucellosis was defined by the clinical findings compatible with brucellosis, positive standard tube agglutination titer (1:160 or higher) and/or isolation of *Brucella *species from blood, bone marrow or tissues [1]. Spinal brucellosis, including spondylitis, sacroiliitis and paraspinal abscess, was diagnosed in the patient with brucellosis who had MRI scan showing skeletal involvement characteristic of osteomyelitis [2].

Brucellosis was categorized according to the length of symptoms as acute (less than 8 weeks), subacute (from 8 to 52 weeks) or chronic (more than 1 year) [9].

Therapeutic failure was considered if symptoms and signs of the disease persisted or increased, with increased MRI findings, at the end of 12 weeks therapy [11].

Relapse was defined by the reappearance of symptoms or signs of the disease or new positive blood cultures during the 12 months after therapy [11].

Sequelae were considered to have occurred when pain, abnormal physical findings or functional limitation persisted for longer than six months posttherapy. The severity of clinical sequelae was classified according to the patient's functional status: normal, no pain or neurological deficits remained; mild sequelae, no neurological deficits remained but pain with exercise that did not interfere with work was present; moderate sequelae, pain interfered with work or milder motor or sensorial deficits remained; and severe sequelae, permanent and excruciating pain (requiring rest and analgesics) or motor or sensorial deficits remained [2].

Adverse effects that limited the therapy were; vomiting for more than three consecutive days despite antiemetic therapy, hepatotoxicity, defined as a >5-fold increase in the aspartate or alanine aminotransferase level over the baseline value or more than 10 times the upper limit of the normal range; nephrotoxicity, defined as more than twice the upper limit of the normal creatinine level range; ototoxicity, serious photosensitivity dermatitis manifested by an exaggerated sunburn reaction, marked erythema with edema or vesiculation or hypersensitivity reactions; central nervous system toxicity, headache, dizziness, insomnia, hallucinations, seizures [12].

Operation indications were; to develop spinal instability, cauda equina syndrome and severe weakness of muscle due to extradural inflammatory mass or collapse of the vertebral body [2,6]. Symptoms and sign that may be indicative of clinical instability include low back pain exacerbated with standing and lifting and relieved by lying down. A sudden catch when extending from the flexed to the straight posture is helpful in identifying instability (21). A stability checklist was also developed for the lumbar spine (Table 1). A point value total of 5 or more indicates clinical instability (22).

**Table 1:** Checklist for the diagnosis of clinical instability in the lumbar spine

**Element**	**Point value**
Anterior elements destroyed or unable to function	2
Posterior elements destroyed or unable to function	2
Radiographic criteria	4
Flexion-extension radiographs	
Sagital plan translation >4.5 mm or 15% (2pt)	
Relative sagittal plane angulation >22 degress (2pt)	
Cauda equine damage	3
Dangerous loading anticipated	1

### Statistical analysis

Data were analysed by Statistical Package for the Social Sciences (SPSS) version 10.0, × ^2 ^test for qualitative variables, and Student's t test for quantitative variables.

## Results

### Patient characteristics

During the study period, a total of 405 patients of brucellosis were diagnosed and followed up in Erciyes University Hospital. Among these patients, 38 (9.4%) were spinal brucellosis. Of these patients, seven patients excluded from the study for different reasons (three patients' therapy was changed by other institution, two patients had adverse reactions, two died because of underlying disease). Thirty-one patients were evaluated in the study. Seventeen (55%) patients were male and 14 (45%) patients were female. The average age was 54.2 ± 17.0 years (range 16–83). Twenty-eight (90%) patients had a history of ingestion of raw milk products and nine (29%) patients had occupational exposure. Brucellosis was acute in 11 patients, subacute in 19 patients and chronic in one patient. The most common symptoms were back pain (90%), malaise (87%) and arthralgia (87%). Only nine patients were hospitalised at the beginning of the therapy, others were followed up in outpatient clinic. Twenty (65%) patients complained about fever, however only four (13%) patients had fever on physical examination. And only three (9.7%) patients had hepatomegaly and one (3%) patient had splenomegaly. Laseque test was positive in 13 patients. Cauda equina syndrome was positive in one patient and severe weakness of muscle in two patients.

Fifteen patients were included in DS group, whereas 16 patients were included in CR group. The demographic and clinical variables of the patients at baseline in the two groups are seen in Table 2. The median age was higher in CR group than DS group (p = 0.007). However there was no difference in gender, occupational exposure was more prevalent in CR group. Furthermore, the duration of symptoms before therapy was not significantly different in both groups (p = 0.768). Twelve week drug-cost was 42 Euro in DS and 50 Euro in CR group in our country.

**Table 2:** Clinical characteristics of 31 patients at baseline in the two groups

Characteristic	**DS (n = 15)**	**CR (n = 16)**
**Median age [yr] (range)**	48 (16–65)	68 (25–83)
**Male (%)**	9 (60)	8 (50)
**Brucellosis risk factor**		
**Occupational exposure (%)**	1 (6.7)	8 (44.4)
**Ingestion of unpasteurized dairy products (%)**	14 (93.3)	14 (87.5)
**Duration of symptoms before therapy (weeks)**		
**Mean ± SD**	14.9 ± 13.0	15.3 ± 12.0
**Median (range)**	12 (2–40)	14 (2–48)
**Operation before therapy n (%)**	0	5 (31)
		
**Symptoms**	**n (%)**	**n (%)**
Malaise	12 (80)	15 (94)
Back pain	14 (93)	14 (88)
Arthralgia	13 (87)	14 (88)
Sweating	11 (73)	15 (94)
Anorexia	7 (47)	15 (89)
Fever	9 (60)	11 (69)
Myalgia	9 (60)	11 (69)
Nausea	4 (27)	5 (31)
Abdominal pain	2 (13)	5 (31)
Vomiting	2 (13)	2 (13)
		
Findings		
**Fever**	1	3
**Hepatomegaly**	2	1
**Splenomegaly**	0	1
**Laseque test positivity**	5	8
		
**Clinical spectrums (duration of symptoms)**		
**Acute (<8 weeks)**	6	5
Subacute (8–52 weeks)	9	10
Chronic (>52 weeks)	0	1
Patients hospitalised	3	6

**Laboratory findings of 31 patients at baseline in the two groups**		

Laboratory finding	**DS (n = 15) (%)**	**CR (n = 16) (%)**


**Anemia**	4 (27)	7 (44)
**Leucocytosis**	0	2 (13)
**Median ESR**	22 (3–110)	54 (2–88)
**Median CRP**		
**Before therapy**	33 (3–151)	53 (3–140)
**After therapy**	3 (3–5)	5 (3–14)
**Median agglutination titer (range)**	320 (160–2560)	320 (160–1280)
**Positive blood culture (%)**	0	4 (22)

### Laboratory findings

The most common laboratory finding was elevated CRP (n = 21, 67.7%) and elevated ESR (n = 19, 61%). Anaemia (haemoglobin <12 mg/dl in female, <14 mg/dl in male) was found in 11 patients and leukocytosis (>10 000/mm^3^) in two patients. Leukopenia and thrombocytopenia were not determined. A moderate elevated hepatic enzymes was found in two patients.

Laboratory findings of the patients at baseline in the two groups are shown in Table 3. The median ESR was significantly higher in CR group before treatment (p = 0.049). However it was not significantly different (p = 0.321), CRP was higher in CR group. Also CRP declined after treatment in the both group.

**Table 3:** Forty-two vertebral level involvement in 31 patients in the two groups

**Characteristic of involvement**	**DS (n = 15)**	**CR (n = 16)**
Multifocal involvement (%)	6 (40)	3 (22)
Abscess formation (%)	4 (26.7)	9 (50)
		
**Vertebral level**		
**Cervical spine (n = 1)**		**1**
C5-6		1
		
**Thoracal spine (n = 6)**	**3**	**3**
T7-8		1
T7-L2		1
T9-10	2	1
T11-12	1	
		
**Lumbar spine (n = 32)**	**16**	**16**
L1-2		3
L2-3	1	4
L3-4	2	3
**L4-5**	**7**	**3**
**L5-S1**	**6**	**3**
		
**Sacroiliitis (n = 3)**	3	

Brucella agglutination titer was ranging between 1/160 and 1/2560) in all patients and there was no difference in the two groups. However, blood cultures were taken in 26 patients and positive only in four (15.4%) patients in CR group. Positive blood culture was more prevalent in CR group.

### Radiological findings

As radiological findings, 42 levels of spinal column were involved in 31 patients. The most common affected site was lumbar spine (n = 32, 76%), followed by thoracic spine (n = 6, 14%), sacroiliac joint (n = 3, 8%) and cervical spine (n = 1, 2%). Involvement level was not different in two groups. Twenty-two (71%) patients had involvement of only a single spinal region (nine in DS group, 13 in CR group), whereas five (16%) patients had contiguous involvement at multiple sites (four in DS group, one in CR group) and four (12.1%) patients had noncontiguous multifocal involvement (two in DS group, two in CR group). Furthermore, 13 (39.3%) patients had abscess formation with vertebral involvement (four patients in DS group and nine patients in CR group). Four (13%) patients had spinal instability due to collapse of vertebral body (Table 4).

**Table 4:** Outcome of the 31 patients in the two groups

	**DS (n = 15)**	**CR (n = 16)**
Duration of therapy		
12 weeks	8	12
14 weeks	3	1
16 weeks	3	2
20 weeks	0	1
24 weeks	1	0
**Therapeutic failure**	0	0
**Operation after therapy (n)**	2	1
**Relapse**	0	0
**Clinical sequelae n (%)**	9 (60)	11 (69)
Mild sequelae	8	9
**Moderate sequelae**	1	2
**Severe sequelae**	0	0

### Treatment and outcome

Two patients discontinued the ciprofloxacin therapy because of dizziness and switched to doxcycline plus rifampicin therapy. No adverse effect was observed in DS group. All the patients had at least 12 weeks antibiotic therapy and bed rest at least two weeks in the both group. Lumbosacral orthosis was suggested to the patients with pain due to instability until resolving symptoms of instability. The median duration of antibiotic therapy was 12 weeks (range 12–24 weeks) in all patients. However therapeutic failure was not observed in any of these patients, 11 patients' therapy was prolonged due to partial clinical and radiological improvement. In DS group, the duration of the therapy was 12 weeks in eight patients, 14 weeks in three patients, 16 weeks in three patients and 24 weeks in one patient and the mean duration was 14.0 ± 3.2 (median = 12, range 12–24). On the other hand, in CR group the duration of the therapy was 12 weeks in 12 patients, 14 weeks in one patient, 16 weeks in two patients and 20 weeks in one patient and the mean duration was 13.1 ± 2.3 (median = 12, range 12–20) (Table 4). The duration of antibiotic therapy was not significantly different in both groups (p = 0.3519).

**Figure 3:**
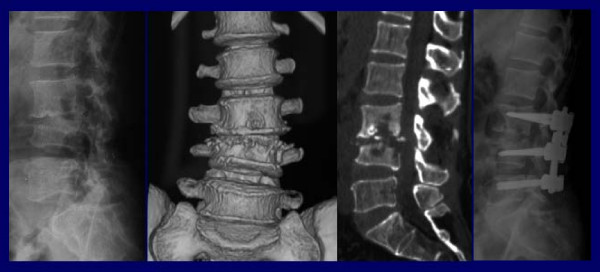
A) Lateral graphy, B) Coronal 3D reconstruction computed tomography, C) Sagittal reformate image: height loss of L4 vertebrae, narrowing disc space with end plates destruction. D) Postoperative lateral graphy: improvement of vertebral axis and disc space.

Eight (25.8%) patients underwent surgical intervention. Five of them underwent operation at the first week of the antibiotic therapy (two for instability -figure 3-, one for cauda equina syndrome, two for severe weakness of muscle) and these patients were all in CR group. On the other hand, three of the patients underwent operation after antibiotic therapy. Two of these patients were in DS group and one of them was operated for instability at the end of the 20 weeks therapy, the other one had 16 weeks therapy and operated three months after the therapy for instability. The other patient was in CR group and operated at the end of 16 weeks therapy for instability.

At the end of the therapy, a good clinical response was observed in 30 patients. However, of these 30 patients, 24 patients (11 in DS group, 13 in CR group) defined mild pain at the end of the therapy. One patient who had operation for instability before and after the therapy defined moderate pain. Furthermore, physical examination findings improved in most of the patients and Laseque test was positive only in four patients. All patients underwent control MRI at the end of the therapy and MRI findings all improved. Patients were phoned 12 months later the therapy and asked about symptoms or signs of the disease. If any symptoms or signs were described, they examined again for motor or sensorial deficit. Relapse was not observed in the both groups, but sequela was observed in 20 (64.5%) patients (nine in DS group, 11 in CR group). The development of sequelae was not significantly different in the two groups (p = 0.716, × ^2 ^= 0.0178) (Table 4).

## Discussion

Spinal brucellosis is one of the most common complication of human brucellosis in endemic regions [1,9]. In this study its incidence was 9.4% and it was 12.3% in our previous study [4]. Ingestion of unpasteurized dairy products (raw milk, butter, cheese) is the most common source of infection [1] and 90% of our patients had a history of ingestion of raw milk and products. Furthermore, brucellosis has been accepted an occupational disease and consequently male gender are frequently affected [1]. In our study each sex is affected similarly. High incidence of ingestion of raw milk products can explain this equivalence.

The symptoms of spinal brucellosis may be insidious and non-specific, especially in subacute and chronic forms [1,9]. In our study, subacute form was more common and back pain, malaise, and arthralgia were the most common complaints. Fever is another common clinical manifestation in spinal brucellosis and undulant fever is described in untreated patients [1,23]. However, 65% of our patients complained about fever, only four patients had fever on physical examination. Since most of our patients were followed up in outpatient clinic, fever may be overlooked in our study.

The diagnosis of spinal brucellosis is very important for the duration of antibiotics but early diagnosis is difficult. Since the early radiological signs of spinal brucellosis are non-specific and may appear late after the onset of symptoms [24]. The appearance of destructive changes on direct radiographs is about three months and computed tomography may not be always helpful in the differential diagnosis from other degenerative diseases and disc herniation [7,25]. MRI is the most useful method for diagnosis, assessment and management of spinal brucellosis. Also it has high sensitivity for differential diagnosis and identifying extent of brucellar spondylitis [26]. Consequently, MRI was used for the diagnosis and assessment in this study. Spinal involvement was observed in forty-one levels and lumbar spine was the most common involved site, particularly L4-L5 and L5-S1 in our patients, as reported in previous studies [2-7]. Moreover, multiple site involvement can be seen and the reported incidence was 9% in the previous studies [2,7]. In our study, multifocal involvement was 30.3% and, moreover, four patients had non-contiguous involvement at multiple sites. Abscess formation has been rarely reported in the past [7], but in recent years, with highly sensitive diagnostic techniques, it becomes a common finding (21%) [6], as in this study (42%). Sacroiliitis is one of the most common form of osteoarticular involvement [4,5], however only 9% of our patients had sacroiliitis.

Despite its frequency in developing countries, the optimal treatment regimen is still controversial. The combination of tetracycline/doxycycline and streptomycin is accepted by the World Health Organization (WHO) as the treatment of choice for spinal brucellosis [1]. However, the reported success rate with this classical regimen is 60%–90%. Moreover, therapeutic failure and relapse rates are still common [2,7]. In our previous report, the relapse rate in osteoarticular involvement was 12.1% and the highest relapse rate was in tetracycline+streptomycin group (14.3%) [4]. Also, the inconvenience of intramuscular streptomycin administration and the toxicity of tetracycline/doxycycline limited the adaptation of patients. The second alternative regimen, the combination of rifampin-doxycycline, has been shown to be less effective than doxcycline-streptomycin [11,12]. As a result, alternative regimens are investigated in recent years. Quinolones are the most commonly studied antibiotics because of their good bactericidal activity and penetration into leukocytes and macrophages. However, monotherapy with these antibiotics showed high relapse rates [27,28], combination regimens with rifampin were found as effective as doxcycline plus rifampin [17,18]. To our knowledge, combination regimens including quinolones were not compared with the classical regimen, doxcycline-streptomycin, in spinal brucellosis. Because of their success in bone infections, they can be alternative as oral regimen to doxcycline-streptomycin therapy in spinal brucellosis. The aim of this study was to compare quinolone-rifampicin efficacy in spinal brucellosis with doxcycline-streptomycin. However, there were differences between the both groups, (patients in CR group were older, the median ESR and CRP were higher at baseline, abscess formation and operation indication before therapy were more prevalent in this group and multifocal involvement was more prevalent in DS group), the outcome was not different in the two groups. Therapeutic failure wasn't observed in the two treatment groups.

The duration of antibiotics in spinal brucellosis is also controversial. As relapse rate and therapeutic failure were significantly higher in shorter durations [1,9], the duration of antibiotics was at least 12 weeks in our study. It was prolonged according to the resolution of clinical and MRI findings. There was no difference in median duration (12 weeks) of therapy in the both groups. In the comparison of the adverse effects, no adverse effect was observed in DS group and two patients in CR group stopped the treatment because of dizziness. Relapse was not observed in any patient after one year of the therapy. Probably, this is the result of appropriate duration of antibiotics.

The diagnostic or curative surgery can be performed in spinal brucellosis but it is last option in the management of spinal brucellosis. The indications for surgery are; spinal instability, cauda equina syndrome, severe weakness of muscle due to extradural inflammatory mass or progressive collapse [6,7]. Eight patients in our study underwent operation and the most common indication for surgery was instability. The need for operation after therapy was not different in the both treatment group. Abscess drainage is not always needed in the absence of neurological deterioration [7] and only one of our patients underwent abscess drainage for neurological deficit; others all improved with medical management.

A delayed convalescence after treatment is the well-known clinical sequela in spinal brucellosis. The reason can't be understood, but pscychoneurosis exacerbated by the infection is believed to be a reason for the delay [1]. Mild clinical sequela was observed equally in the both group one year after the therapy. Only one patient in CR group who had operation before and after the therapy had moderate sequale.

The reported disadvantage of quinolone including is the high cost rates [18,19]. In this study we only calculated the drug cost for the median duration (12 weeks) of the both group and the cost of CR therapy was 1.2 fold higher than the cost of DS therapy.

## Conclusion

Ciprofloxacin-rifampicin regimen is as effective as classical regimen. Despite the disadvantages (older age, more prevalent operation and abscess formation before the therapy) of the patients in CR group, the duration of the therapy and clinical response were not different from the classical regimen. So combination of ciprofloxacin and rifampin can be an alternative regimen in spinal brucellosis. However it can't be considered as first-line therapy because of its high cost that is important for developing countries. Classical regimen (doxycycline with streptomycin), with the appropriate duration (at least 12 weeks), is still the first line antibiotics and alternative therapies should be considered when adverse reactions observed.

## Abbreviations

Ciprofloxacin C

Rifampicin R

Doxycycline D

Streptomycin S

Magnetic resonance imaging MRI

Erythrocyte sedimentation rate ESR

C reactive protein CRP

Statistical Package for the Social Sciences SPSS

## Competing interests

The author(s) declare that they have no competing interests.

## Authors' contributions

**EA **followed the patients and drafted the manuscript.

**RKK **followed the patients and performed the operations.

**ACD **evaluated magnetic resonance imaging.

**OY **and **BA **participated in the design of the study.

**BS **evaluated the microbiological results.

**MD **participated in the design of the study and coordination, and involved in drafting the manuscript.

## Pre-publication history

The pre-publication history for this paper can be accessed here:


